# Psychometric Properties of the Chinese SUPPS-P Impulsive Behavior Scale: Factor Structure and Measurement Invariance Across Gender and Age

**DOI:** 10.3389/fpsyt.2020.529949

**Published:** 2020-11-19

**Authors:** Yingying Wang, Jiang Long, Yueheng Liu, Tieqiao Liu, Joël Billieux

**Affiliations:** ^1^Department of Psychiatry, The Second Xiangya Hospital, Central South University, Changsha, China; ^2^Hunan Key Laboratory of Psychiatry and Mental Health, The China National Clinical Research Center on Mental Health Disorders (Xiangya), Chinese National Technology Institute on Mental Disorders, Mental Health Institute of Central South University, Changsha, China; ^3^Shanghai Mental Health Center, Shanghai Jiao Tong University School of Medicine, Shanghai, China; ^4^Laboratory for Experimental Psychopathology, Psychological Science Research Institute, Université Catholique de Louvain, Louvain-la-Neuve, Belgium; ^5^Institute of Psychology, University of Lausanne, Lausanne, Switzerland

**Keywords:** problematic behaviors, measurement invariance, psychometric properties, UPPS, impulsivity, short form

## Abstract

**Objective:** Impulsivity is widely recognized as a risk factor for a variety of mental disorders and problematic behaviors. The Short UPPS-P Impulsive Behavior Scale (SUPPS-P) is an extensively used instrument to measure impulsivity in research and clinical settings. The current study primarily aimed to evaluate the psychometric properties of the Chinese version of the SUPPS-P (C-SUPPS-P) among Chinese adolescents and emerging adults, and then to test its measurement invariance across gender and age.

**Methods:** Data were collected from three vocational high schools and six colleges in Changsha, China. A total of 2,551 participants (20.1% male and 22.6% adolescents) completed the C-SUPPS-P and scales assessing addictive and problematic smartphone use, as well as emotional symptoms (anxiety, stress, depression). Four alternative models were examined and compared by using confirmatory factor analysis to determine the best factor structure of the C-SUPPS-P. Multigroup confirmatory factor analyses were used to test measurement invariance across gender and age.

**Results:** A theory-driven five-factor structure consistent with the original scale was identified. All of the subscales had good internal consistency. The correlations observed with the other scales supported the construct validity of the C-SUPPS-P. Full measurement invariance was established across gender and age, and significant gender and age differences according to impulsivity facets were identified.

**Conclusions:** The C-SUPPS-P presents a consistent factor structure, as well as reliability and validity that are equivalent to those of the original scale. The full measurement invariance shown across gender and age allows for intergroup comparisons. Overall, the C-SUPPS-P is a promising instrument to measure various impulsivity traits in Chinese adolescents and emerging adults.

## Introduction

Impulsivity as a personality trait is a central construct in the field of psychopathology and neuropsychology. Impulsivity is also used as a common diagnostic criterion in internationally accepted diagnostic systems, e.g., the *Diagnostic and Statistical Manual of Mental Disorders*, 5th edition (1). The numerous mental conditions for which impulsivity is a diagnostic feature include borderline personality disorder, antisocial personality disorder, attention deficit hyperactivity disorder, conduct disorder, bipolar disorder, and substance use and addictive disorders. Impulsivity is unanimously considered to be a multifaceted construct encompassing various distinct but interrelated psychological dimensions (2, 3). However, the debate over the number of its dimensions continues to persist, ranging from two-factor models to models encompassing more than 10 different dimensions (4). Accordingly, numerous scales have been developed to assess impulsivity-related traits. In the last decade, a scale that has gained attention and is increasingly used is the UPPS-P Impulsive Behavior Scale (UPPS-P) (5), which was created on the basis of the impulsivity model proposed by Whiteside and Lynam (3).

Whiteside and Lynam (3) conducted a seminal study in which they capitalized on all major impulsivity scales and the Big Five traits (6) in order to develop a new integrative measure: The UPPS Impulsive Behavior Scale (UPPS) (7). This scale initially comprised four dimensions of impulsivity, consisting of negative urgency (defined as the tendency to act rashly in reaction to intense negative affect), lack of premeditation (defined as the tendency not to take into account future consequences of an action), lack of perseverance (defined as the tendency not to focus on demanding and/or boring tasks), and sensation seeking (defined as the openness to new experiences and the tendency to engage in risky and stimulating actions). The UPPS has since been validated in various languages, including English (8), French (9), and German (10). Subsequently, Cyders and colleagues (11, 12) identified a positive urgency impulsivity component (defined as the tendency to act rashly in reaction to intense positive affect), which resulted in an updated scale: the UPPS-P (5). This updated scale has shown good psychometric properties in various languages and regions (13–16), as well as measurement invariance across gender, age groups, and ethnicity (17–19). In recent years, a growing number of studies conducted in both clinical and community samples have used the UPPS-P to explore the role of distinct impulsivity components in various problematic behaviors and psychopathological symptoms in both clinical and community samples (20, 21), such as substance abuse (22–24), pathological gambling (25–28), problematic mobile phone use (29, 30), or risky sexual behaviors (31, 32). The UPPS-P can be considered a theoretically and psychometrically sound tool to assess the multidimensional impulsivity construct; nevertheless, its main limitation is its length (59 items) and time-consuming aspects (~10–15 min). This issue is all the more problematic when the UPPS-P is used with people who are cognitively impaired, fatigable, or young. To counter this important limitation, short forms of the UPPS-P have been developed and validated.

Two versions of the Short UPPS-P (SUPPS-P) are extensively used (four items per subscale for a total of 20 items): one was first developed in French by Billieux et al. (33) and the other was later developed in English by Cyders et al. (34). The main difference between these two versions is the approach used for item selection. Specifically, Billieux et al. (33) selected items that loaded highest on each corresponding impulsivity facet from the original French UPPS Impulsive Behavior Scale (35) and the Positive Urgency Measure (12). In contrast, Cyders and colleagues selected items with the highest corrected item-total correlations in each original subscale of the original English scale (5). Despite methodological differences in item selection, numerous studies have shown that both versions have a solid and theoretically sound factor structure in diverse languages. Short versions of the scale developed by Billieux et al. (33) have, for example, been developed and validated in Spanish (36), Italian (37), Arabic (38), and Hungarian (39), whereas the version proposed by Cyders et al. (34) has been validated in Farsi (40), Swedish (41), Korean (42), and Portuguese (43)^1^. It is worth stressing that studies also confirmed that the short versions were psychometrically equivalent to the full UPPS-P, but greatly decreased the evaluation time (33, 34, 42).

The current study aimed to develop and validate a Chinese short version of the UPPS-P (C-SUPPS-P) on the basis of the original SUPPS-P (33), which has proven to be an effective tool for assessing the impulsivity of both the general population and clinical patients (44–47). To achieve this objective, we conducted a validation study in a sample of Chinese adolescents and emerging adults, a group that is prone to impulsivity for several reasons including immaturity of the brain areas involved in self-control and self-regulation (48, 49). The factor structure of the C-SUPPS-P was explored through comparison of various models by using confirmatory factor analysis (CFA). The construct validity of the C-SUPPS-P was approached through the exploration of its specific relationships with emotional symptoms (anxiety, depression, and stress) and with problematic and addictive use of the smartphone, which is a prevalent issue in mainland China (50). Given that many studies have found age- and gender-related differences in impulsivity traits (17, 18), our second objective was to test the measurement invariance of the C-SUPPS-P across gender and age.

## Materials and Methods

### Participants

We conducted an online survey at nine schools (three vocational high schools and six undergraduate schools) in Changsha, China. The subjects volunteered to participate in this study after the class counselors forwarded the survey link. A total of 2,555 individuals completed all of the questionnaires used in the current study. We excluded four participants who were outliers regarding age and retained a final sample of 2,551 participants, including 513 (20.1%) males and 2,038 (79.9%) females, 576 of whom (22.6%) were below age 18 years and 1,975 of whom (77.4%) were above age 18. The mean (±SD) age was 18.38 (±1.58) for the total sample, 18.32 (±1.60) for males, and 18.62 (±1.49) for females.

Before completing the study, all participants provided online informed consent. For participants under the age of 18, online informed consent was obtained from their parents (or legal guardians). The study protocol was approved by the Ethics Committee of the Second Xiangya Hospital of Central South University. Some measures included in the online survey were unrelated to the current study and will be presented elsewhere. Part of the data described here were used to test the psychometric properties of the Problematic Mobile Phone Use Questionnaire—Short Version [PMPUQ-SV, (51)].

### Instruments

#### The Chinese Version of the SUPPS-P (C-SUPPS-P)

The SUPPS-P Impulsive Scale (33) assesses five facets of impulsivity (four items in each dimension): negative urgency, lack of premeditation, lack of perseverance, sensation seeking, and positive urgency. Items are scored on a 4-point Likert scale (some items have to be reversed before scoring), with a high score reflecting high impulsivity. We translated the original French items into Chinese according to the standard scale revision procedure (52) and then conducted a back-translation. This process was performed by two authors (JL and YL) who are proficient in Chinese and English and another author (JB) who is proficient in French and English and who is also the developer of the original SUPPS-P. All discrepancies identified in the translated items were discussed until a satisfactory solution was found.

#### The Chinese Version of the Smartphone Addiction Proneness Scale (C-SAPS)

The 15-item SAPS (53) is a self-reported scale that aims to assess the symptoms and severity of addictive smartphone use (i.e., loss of control, withdrawal, tolerance, and online life orientation). The SAPS was translated into Chinese in the current study. The C-SAPS adopted a 4-point Likert score, high scores indicating more serious addictive smartphone use. In the current study, the total score of the C-SAPS was used as a measure of addictive smartphone use. Cronbach's α of the full scale was 0.850.

#### Dangerous Use (DU) Subscale of the PMPUQ-SV

We used the DU subscale of the Chinese PMPUQ-SV (51) to assess risky smartphone use behaviors. The full C-PMPUQ-SV was adapted from the study by Lopez-Fernandez et al. (54). Only the DU subscale was used in the current study, as addictive use of the smartphone was covered by the C-SAPS. Items were scored from 1 (“I strongly agree”) to 4 (“I strongly disagree”) (some items have to be reversed before scoring), with higher scores indicating more dangerous smartphone use (e.g., use of a smartphone while driving, riding, or crossing the road). In the current study, Cronbach's α for the DU subscale was 0.768.

#### Depression Anxiety Stress Scales-21

These scales (55, 56) were designed to measure three types of emotional symptoms related to depression, anxiety, and stress. Items are scored on a 4-point Likert scale ranging from 0 (did not apply to me at all) to 3 (applied to me very much, or most of the time), with higher sum scores for each subscale indicating more serious emotional symptoms. Items focus on emotional symptoms that participants experienced in the past week. The Chinese version of the scale presents with good psychometric properties (57). In the current study, Cronbach's α values for the depression, anxiety, and stress subscales were 0.872, 0.773, and 0.838, respectively.

### Data Analysis Procedure

We performed statistical analysis by using SPSS 23.0 software (IBM, Armonk, NY) and Mplus 7.4 (Muthén & Muthén, Los Angeles, CA) in accordance with the following four steps.

#### Item Analysis and CFA

Item analysis consisted of computing the item-total and corrected item-total correlations to determine whether each item can be used as an effective indicator to measure the targeted impulsivity facet. Four specified models, corresponding to the models tested in previous validation studies (33, 36), were computed and compared in order to determine the best factor structure for the C-SUPPS-P. Model 1 specified that all items constitute a single and unitary impulsivity factor. Model 2 was a three-factor model, in which the first factor corresponded to urgency and consisted of items of both negative and positive urgency; the second factor was labeled “deficit in conscientiousness” [in accordance with the proposition made by Cyders and Smith (58)], which grouped items for lack of premeditation and lack of perseverance facets into a single factor; and the third factor corresponded to sensation-seeking items. Model 3 represented a one-order structure with five distinct but internally related dimensions. Model 4 consisted of a hierarchical model wherein urgency was set as a higher order factor to account for positive and negative urgency, deficit in conscientiousness [in accordance with the proposition made by Cyders and Smith (58)] was set as another hierarchical factor to account for lack of premeditation and lack of perseverance, and sensation seeking constituted a separate first-order factor. According to the modification indices, the residuals of two pairs of items (Items 5 and 8; Items 2 and 4) were allowed to covary, as they were composed of items that were very close at the semantic level. Multiple fit indices were used to assess model fit, including the comparative fit index (CFI), the root mean-square error of approximation (RMSEA), and the standardized root mean square residual (SRMR). The criteria used to define an acceptable model were CFI ≥ 0.90 and RMSEA and SRMR ≤ 0.08 (59). We used Satorra-Bentler's Maximum Likelihood Mean Adjusted estimator, as it is suitable for a slightly non-normal distribution (the skewness ranged from 0.034 to 0.391 and the kurtosis ranged from 0.186 to 1.054 in the current study). Notably, we considered the chi-square as a reference but not a criterion because it is easily dependent on sample size (60).

#### Internal Consistency and Construct Validity

Internal consistency of the various impulsivity subscales was tested with Cronbach's alpha. Then, to establish construct validity, we calculated the two-tailed Pearson correlation coefficients between the C-SUPPS-P and the other measures used to measure addictive and problematic mobile phone use, as well as emotional symptoms.

#### Measurement Invariance Across Gender and Age

We tested measurement invariance of the best fit model of the C-SUPPS across gender (male and female) and age (<18 years and ≥18 years). A sequential strategy as proposed by Meredith and Teresi (61) was used to perform these tests. First, configural invariance indicates a similar pattern of factor construction between the two comparable groups; that is, the path plot of the factor model appears similar across groups. Second, metric invariance (or weak invariance) implies that factor loadings are equivalent between groups. Third, scalar invariance (or strong invariance) implies that the intercepts of observed variables have equivalence between different groups. Finally, the last and strictest step corresponds to error variance invariance (or strict invariance), which implies that the error variance of the latent variables is to be equal across groups. As the difference derived from the chi-square test was hypersensitive to the increase in sample size, we adopted the difference in CFI, RMSEA, and SRMR (i.e., ΔCFI, ΔRMSEA, and ΔSRMR) to evaluate the equivalence, and a Δ value of ≤ 0.01 was considered acceptable (62).

#### Gender and Age Difference Test

Following the identification of the measurement invariance, a series of independent sample *t-*tests were computed in order to examine gender and age differences with regard to the various impulsivity traits.

## Results

### Item Analysis and CFA

Item analysis found that Item 11 (from the lack of perseverance subscale) was not correlated with the total score of 20 items (*r* = 0.026) and was negatively correlated with the corrected total score (*r* = −0.088; see Supplementary Table 2); thus, we eliminated it for subsequent CFAs. As shown in Table 1, the single-factor model (Model 1) had a poor fit, confirming that impulsivity constitutes a multidimensional rather than a single structure. The three-factor model (Model 2) was slightly better than the single-factor one, but also failed to reach an acceptable fit. Both the five-factor model (Model 3) and the hierarchical model (Model 4) fit the data well (mainly manifested in the RMSEA and SRMR values, which were sensitive to the misspecification of the factors loading and covariance, both being close to 0.5). As Model 4 specified less covariance between factors, it was slightly more parsimonious in structure than Model 3 was. However, the chi-square difference test (Δχ^2^ = 13.58, Δ*df* = 3, *p* < 0.01) showed that Model 3 fit the data significantly better than Model 4 did. From this consideration, we finally retained Model 3, which consists of a single-order model composed of five specific but interrelated impulsivity dimensions.

**Table 1:** Comparison of goodness-of-fit indices and models (*n* = 2,551).

**Model**	**S-Bχ^2^**	***df***	***p***	**CFI**	**RMSEA (90% CI)**	**SRMR**
1	4890.531	150	<0.001	0.611	0.111 (0.109–0.114)	0.133
2	1396.809	147	<0.001	0.898	0.058 (0.055–0.061)	0.056
3	1169.740	140	<0.001	0.916	0.054 (0.051–0.057)	0.050
4	1181.898	143	<0.001	0.915	0.053 (0.051–0.056)	0.052

*S-Bχ^2^, Satorra-Bentler corrected chi-square; df, degrees of freedom; CFI, comparative fit index; RMSEA, root mean square error of approximation; CI, confidence interval; SRMR, standardized root mean square residual; Model 1, a single factor model; Model 2, a three-factor model, including urgency, conscientiousness, and sensation seeking; Model 3, a five-factor one-order model; Model 4, a five-factor hierarchical model*.

### Construct Validity and Internal Consistency Reliability

To examine construct validity, we considered Pearson's correlations between the five impulsivity dimensions and the external correlates (addictive smartphone use, dangerous use of the smartphone, depression, anxiety, and stress). Table 2 shows that negative urgency was moderately associated with stress, anxiety, depression, and smartphone addiction (all correlations between *r* = 0.30 and *r* = 0.50) and weakly correlated with dangerous use of the smartphone (*r* = 0.24). Lack of perseverance was weakly correlated with depression, stress, and smartphone addiction (all correlations between *r* = 0.10 and *r* = 0.30). Sensation seeking was weakly correlated with anxiety, depression, stress, and smartphone addiction (all correlations between *r* = 0.10 and *r* = 0.30). Positive urgency was moderately correlated with stress (*r* = 0.34) and weakly correlated with depression, anxiety, smartphone addiction, and dangerous use of the smartphone (all correlations between *r* = 0.10 and *r* = 0.30). Correlations for which *r* < 0.10 were not considered relevant even if they were statistically significant due to sample size.

**Table 2:** Internal consistency and construct validity (*n* = 2,551).

	**Measure**	**1**	**2**	**3**	**4**	**5**	**6**	**7**	**8**	**9**	**10**
1	NU	(0.734)									
2	PR	−0.062^**^	(0.740)								
3	PE	−0.005	0.604^**^	(0.773)							
4	SS	0.379^**^	−0.263^**^	−0.192^**^	(0.741)						
5	PU	0.664^**^	−0.118^**^	−0.048^*^	0.521^**^	(0.702)					
6	C-SAPS	0.329^**^	0.082^**^	0.175^**^	0.082^**^	0.296^**^	(0.850)				
7	DU	0.244^**^	0.048^*^	0.090^**^	0.160^**^	0.237^**^	0.417^**^	(0.768)			
8	Depression	0.347^**^	0.060^**^	0.183^**^	0.184^**^	0.286^**^	0.284^**^	0.220^**^	(0.872)		
9	Anxiety	0.353^**^	−0.014	0.057^**^	0.204^**^	0.297^**^	0.249^**^	0.195^**^	0.658^**^	(0.773)	
10	Stress	0.428^**^	0.012	0.124^**^	0.180^**^	0.336^**^	0.328^**^	0.255^**^	0.760^**^	0.721^**^	(0.838)

* *p < 0.05*.

** *p < 0.01*.

*NU, Negative Urgency; PR, (Lack of) Premeditation; PE, (Lack of) Perseverance; SS, Sensation Seeking; PU, Positive Urgency; C-SAPS, Chinese version of Smartphone Addiction Proneness Scale; DU, Dangerous Use subscale of the Problematic Use of Mobile Phone Questionnaire-Short Version; The values in brackets represent the Cronbach's a of each scale*.

Cronbach's α values (see values in parentheses in Table 2) of the total scale and the five subscales were 0.757 (total scale), 0.734 (negative urgency), 0.740 (lack of premeditation), 0.773 (lack of perseverance), 0.741 (sensation seeking), and 0.702 (positive urgency), implying that all C-SUPPS-P facets have good internal reliability.

### Measurement Invariance and Age/Gender Comparisons

Table 3 depicts the four nested tests of measurement invariance (configural, metric, scalar, and error variance invariance, in order) across gender and age. Given that the fit indices that we focused on (i.e., ΔCFI, ΔRMSEA, and ΔSRMR) all had a difference of < 0.01 at each step, the full measurement invariance was established across gender and age.

**Table 3:** Multiple fit indices for measurement invariance across gender and age (*n* = 2,551).

	**S-Bχ^2^**	***df***	**CFI**	**RMSEA (90% CI)**	**SRMR**	**Δχ^2^**	**Δ*df***	**ΔCFI**	**ΔRMSEA**	**ΔSRMR**
**Gender**
Model A_1_	1315.960^*^	280	0.915	0.054 (0.051–0.057)	0.053	-	-	-	-	-
Model B_1_	1337.324^*^	294	0.915	0.053 (0.050–0.056)	0.053	4.989	14	0.000	0.001	0.000
Model C_1_	1394.379^*^	308	0.911	0.053 (0.050–0.055)	0.054	55.291	14	0.004	0.000	0.001
Model D_1_	1442.889^*^	327	0.909	0.052 (0.049–0.054)	0.054	58.762	19	0.002	0.001	0.000
**Age**
Model A_2_	1294.332^*^	280	0.917	0.053 (0.050–0.056)	0.052	-	-	-	-	-
Model B_2_	1320.141^*^	294	0.916	0.052 (0.049–0.055)	0.053	14.438	14	0.001	0.001	0.001
Model C_2_	1349.631^*^	308	0.915	0.051 (0.049–0.054)	0.053	20.397	14	0.001	0.000	0.000
Model D_2_	1390.608^*^	327	0.913	0.050 (0.048–0.053)	0.053	42.315	19	0.002	0.001	0.000

* *p < 0.001*.

*S-Bχ^2^, Satorra-Bentler corrected chi-square; df, degrees of freedom; CFI, comparative fit index; RMSEA, root mean square error of approximation; CI, confidence interval; SRMR, standardized root mean square residual*.

From the measurement invariance, we tested gender and age differences for all impulsivity traits. As shown in Table 4, men scored significantly higher than women did in sensation seeking (*p* < 0.001), but no significant gender differences were found with regard to the other impulsivity facets. Regarding the age difference, minors (<18 years) scored significantly higher than emerging adults did on all impulsivity facets except sensation seeking.

**Table 4:** Scores (*M* ± SD) on the total C-SUPPS-P and five subscales by gender and age.

	**C-SUPPS-P**	**NU**	**PR**	**PE**	**SS**	**PU**
Female	43.76 ± 6.79	9.60 ± 2.52	9.10 ± 2.32	6.80 ± 1.90	8.97 ± 2.62	9.29 ± 2.41
Male	44.02 ± 6.79	9.65 ± 2.60	8.93 ± 2.60	6.66 ± 2.12	9.48 ± 2.57	9.30 ± 2.51
*t*	−0.769	−0.357	1.287	1.462	−3.958	−0.126
*p*	0.442	0.721	0.198	0.144	<0.001	0.900
Age <18	44.69 ± 6.90	9.83 ± 2.52	9.31 ± 2.30	6.95 ± 1.86	9.08 ± 2.66	9.51 ± 2.46
Age ≥ 18	43.56 ± 6.74	9.55 ± 2.53	8.99 ± 2.40	6.72 ± 1.96	9.07 ± 2.60	9.22 ± 2.41
*t*	3.512	2.372	2.840	2.461	0.060	2.522
*p*	<0.001	0.018	0.005	0.014	0.952	0.012

C-SUPPS-P, Chinese version of the Short UPPS-P Impulsive Behavior Scale; NU, Negative Urgency; PR, (Lack of) Premeditation; PE, (Lack of) Perseverance; SS, Sensation Seeking; PU, Positive Urgency.

## Discussion

The current study was the first to test the psychometric properties of a Chinese version of the SUPPS-P. Similar to what was found in previous studies, in the present study, CFAs showed that a model holding five distinct but interrelated impulsivity components fit the data well. All impulsivity scales were found to have adequate internal reliability, and correlations with emotional symptoms and problematic/addictive usage of the smartphone supported construct validity of the various impulsivity facets assessed. In addition, this study was the first to ascertain measurement invariance of the SUPPS-P across gender and age, further establishing the psychometric properties of this scale.

In comparing various models with CFAs, we confirmed that the C-SUPPS-P has a sound and theoretically driven factor structure embracing five distinct but interrelated facets. Our findings are aligned with those obtained in other validation studies of the SUPPS-P, including the Spanish (36), Italian (37), Hungarian (39), and Arabic (38) versions. Unlike the original study by Billieux et al. (33) holding that a hierarchical model was best to account for the factorial structure of the SUPPS-P (Model 4), the present study showed that a non-hierarchical model (Model 3) fits the data better. Notably, most previous studies also suggested retaining similar non-hierarchical models (36–39). Furthermore, even those authors who retained a hierarchical model defended the position that the five impulsivity constructs should be assessed separately for a fine-grained clinical assessment (33). This later point is particularly important when it comes to assess the effect of a treatment (e.g., medication, psychological intervention) on a specific impulsivity facet. All impulsivity facets reached a Cronbach's α of >0.70, which corresponds to good internal reliability. It is worth noting that preliminary item analysis identified one item (Item 11, assessing lack of perseverance: “Once I start a project, I almost always finish it”) as inappropriate, as this item was largely uncorrelated with the total scores of the scale. It might be that the Chinese translation of this item altered somewhat its meaning so that the item taps on constructs related to motivation or task difficulty, which might not be the case of others lack of perseverance items. After consultation with two additional external Chinese researchers specialized in psychometrics, we came to the conclusion that problematic Item 11 might be unable to truly measure lack of perseverance in the Chinese context due to psycholinguistic factors (e.g., subtle but important change in meaning), and we decided to remove it from the final scale.

The correlations between the C-SUPPS-P subscales and other variables were calculated to examine its construct validity. Results showed differential relationships between the various impulsivity facets and emotional symptoms. First, and unsurprisingly, urgency (both positive and negative) had the strongest relationship with depression, anxiety, and stress, which is consistent with the results of earlier studies (33, 41). This finding suggests that emotion-driven impulsivity may predict behaviors caused by emotional maladjustment, which is in accordance with previous studies that used the original long versions of the UPPS or the UPPS-P and linked this impulsivity facet with problematic behaviors such as dysregulated eating and self-injury (63–67). Although urgency was also associated with smartphone addiction and risky smartphone use, it had a closer relationship in our study with emotional symptoms. Interestingly, when comparing the relationships between the other impulsivity facets with two types of problematic smartphone use (addictive vs. risky smartphone use), we found that lack of perseverance was closely related to smartphone addiction symptoms, whereas sensation seeking was more closely related to risky smartphone use, which was similar to what was found in a previous study by Billieux et al. (30).

Another objective of our study was to establish measurement invariance of the C-SUPPS-P across gender and age. Notably, the computed fit indices difference (all ΔCFI, ΔRMSEA, and ΔSRMR <0.01) supported full measurement invariance across gender and age, which was consistent with what was found for the full version of the UPPS-P (17, 18), indicating that both long and short versions allow for gender and age comparisons. Regarding gender, men scored significantly higher on sensation seeking than women did, which was consistent with the results of previous studies (33, 68–70). Regarding age, participants <18 years of age reported higher scores in all impulsivity facets except sensation seeking. This general heightened impulsivity in adolescents could be explained by neurodevelopmental factors (71–73), as adolescents are emotionally more unstable and often lack of self-regulation skills, which puts them at higher risk of displaying impulsive and problematic behaviors. This supports the relevance of targeting this age group with preventive actions and promotion of early interventions in adolescents with the most pronounced impulsivity traits.

Some limitations of the study must be acknowledged. First, we focused only on adolescents and emerging adults, and thus our results are not representative of the general population. Second, our study is characterized by an unbalanced gender ratio. Fortunately, the male sample size (*n* = 513) was sufficient for the type of data analytic strategy applied. Third, our study design was of a cross-sectional nature, which hindered us from considering the test-retest stability of the C-SUPPS-P. Finally, our sample was only composed of healthy participants and the psychometric properties of the C-SUPPS-P should be confirmed in clinical population (e.g., individuals with substance abuse and addictive disorders). Yet, previous work conducted in other languages suggests that the SUPPS-P supported the psychometric validity of this scale in psychiatric patients encountered in an emergency setting (74) and in substance use disorder patients (45) implying that this short impulsivity scale can be filled by unstable psychiatric patients.

Overall, the C-SUPPS-P presents with a theoretically sound factor structure, its construct validity is supported by specific links with problematic smartphone use and emotional symptoms, and its various facets are characterized by good internal reliability and measurement invariance across gender and age groups (adolescents and emerging adults). Despite further research being necessary to explore its psychometric properties in older groups and in clinical samples, the C-SUPPS-P constitutes a promising tool to measure the multidimensional impulsivity construct in Chinese emerging adults and adolescents.

## Data Availability Statement

The datasets generated for this study are available on request to the corresponding author.

## Ethics Statement

The studies involving human participants were reviewed and approved by the Ethics Committee of the Second Xiangya Hospital of Central South University. Before completing the study, all participants provided online informed consent. For participants under the age of 18, online informed consent was obtained from their parents (or legal guardians).

## Author Contributions

TL, JB, and JL designed the study and supervised the whole process. YW managed the literature search, participated in data collection, and computed statistical analyses. YW and JB wrote the manuscript. JL and YL translated and back-translated the original scale under the supervision of JB. All co-authors have approved the final manuscript.

## Conflict of Interest

The authors declare that the research was conducted in the absence of any commercial or financial relationships that could be construed as a potential conflict of interest.

## Footnotes

^1^The online Supplementary Material contains Supplementary Table 1, which summarizes the various validated versions of the SUPPS-P.

## Supplementary Material

The online Supplementary Material contains Supplementary Table 1, which summarizes the various validated versions of the SUPPS-P, Supplementary Table 2, which shows the results of item analysis and Supplementary Tables 3,4, in which both the French and Chinese version scales are listed.
