# Supplementary material for: Psychometric Properties of the Chinese SUPPS-P Impulsive Behavior Scale: Factor Structure and Measurement Invariance Across Gender and Age
Source: Front Psychiatry. 2020 Nov 19;11:529949. doi: 10.3389/fpsyt.2020.529949 (PMC7710909; doi:10.3389/fpsyt.2020.529949)
Supplement: Supplementary file 1 [file Table_1.DOCX]

**Supplemental Table 1**

Overview of validated versions of the SUPPS-P

| Language | Year | Item sources | Construct validity | Internal consistency reliability (Cronbach's α) |
| --- | --- | --- | --- | --- |
| French | 2012 | 1. Items for NU, PR, PE, and SS were selected from the French UPPS items having the highest loadings on each factor. 2. Items for PU were selected from a translated French version of the PUM having the highest loadings on this factor. | Sex  AUDIT  Spielberger Trait-Anxiety Inventory  BDI-2 | Ranging from 0.70 (PU) to 0.84 (PE) |
| Spanish | 2012 | All items were selected from the Spanish UPPS-P corresponding to the French SUPPS-P. | Emotion Regulation Questionnaire | Ranging from 0.61 (PU) to 0.81 (SS) |
| English | 2014 | All items were selected from the English UPPS-P that had the highest corrected item-total correlation (for each impulsivity facet). | AUDIT  The National Survey on Drug Use and Health Instrument-Modified  Eating Disorder Diagnostic Scale  The South Oaks Gambling Screen  The Self-Harm Inventory | Ranging from 0.74 (SS) to 0.85 (PU and PR) |
| Italian | 2015 | Translated from the French SUPP-S. | Fagerström Test for Nicotine Dependence  CIUS  BDI-2 | Ranging from 0.73 (PR) to 0.84 (PE) |
| Farsi | 2016 | Translated from the English SUPP-S. | Problematic and Risky Internet Use Screening Scale | Ranging from 0.67 (PR) to 0.80 (NU) |
| Arabic | 2017 | Translated from the French SUPP-S. | CIUS | Ranging from 0.58 (PR) to 0.72 (PE) |
| Swedish | 2017 | 1. Items for NU, PR, PE, and SS were selected from the Swedish version of the UPPS corresponding to the English SUPPS-P. 2. Items for PU were translated from the English SUPPS-P. | Depression Anxiety Stress Scale along with two questions:   1. How often did the participants drink alcohol? 2. Did the participants use other substances besides alcohol? | Ranging from 0.60 (PE) to 0.78 (PU and PR) |
| Korean | 2018 | All items were selected from the Korean UPPS-P corresponding to the English version of the SUPPS-P. | BDI-2  State-Trait Anxiety Inventory  Eating Disorder Inventory-2  AUDIT  Canadian Problem Gambling Index | Ranging from 0.65 (PR) to 0.76 (PU) |
| Portuguese | 2018 | 1. Items for NU, PR, PE, and SS were selected from the Portuguese UPPS corresponding to the English SUPPS-P. 2. Items for PU were selected from the Portuguese PUM. |  | Ranging from 0.797 (PE) to 0.875 (PU)^a^ |
| Hungarian | 2019 | Translated from the French SUPP-S. |  |  |

SUPPS-P = Short UPPS-P Impulsive Behavior Scale; NU = Negative Urgency; PR = (Lack of) Premeditation; PE = (Lack of) Perseverance; SS = Sensation Seeking; PU = Positive Urgency; PUM = Positive Urgency Measure; AUDIT = Alcohol Use Disorder Identification Test; BID-2 = Beck Depression Inventory; CIUS = Compulsive Internet Use Scale.

^a^Lucke’s omega, not Cronbach's α.
