# Supplementary material for: Psychometric Properties of the Chinese SUPPS-P Impulsive Behavior Scale: Factor Structure and Measurement Invariance Across Gender and Age
Source: Front Psychiatry. 2020 Nov 19;11:529949. doi: 10.3389/fpsyt.2020.529949 (PMC7710909; doi:10.3389/fpsyt.2020.529949)
Supplement: Supplementary file 2 [file Table_2.DOCX]

**Supplemental Table 2**

Item-total and corrected item-total correlations

| Item | Item-total correlation | Corrected item-total correlation | Item | Item-total correlation | Corrected item-total correlation |
| --- | --- | --- | --- | --- | --- |
|  | | |  | | |
| Negative Urgency | | | Lack of Perseverance | | |
|  | | |  | | |
| 4 | 0.594^**^ | 0.501 | 5 | 0.412^**^ | 0.307 |
| 7 | 0.431^**^ | 0.321 | 8 | 0.414^**^ | 0.315 |
| 12 | 0.575^**^ | 0.483 | 11 | **0.026** | **-0.088** |
| 17 | 0.513^**^ | 0.413 | 16 | 0.285^**^ | 0.179 |
|  | | |  | | |
| Lack of Premeditation | | | Sensation Seeking | | |
|  | | |  | | |
| 1 | 0.367^**^ | 0.260 | 3 | 0.501^**^ | 0.392 |
| 6 | 0.283^**^ | 0.167 | 9 | 0.262^**^ | 0.135 |
| 13 | 0.297^**^ | 0.187 | 14 | 0.335^**^ | 0.217 |
| 19 | 0.252^**^ | 0.138 | 18 | 0.489^**^ | 0.382 |
|  | | |  | | |
| Positive Urgency | | | | | |
|  | | | | | |
| 2 | 0.519^**^ | 0.421 | 15 | 0.564^**^ | 0.476 |
| 10 | 0.498^**^ | 0.395 | 20 | 0.513^**^ | 0.408 |

***p*< 0.01

Underlined letters indicate the initials of each dimension of the UPPS-P. The item-total correlation and corrected item-total correlation of item11 shown in bold.
