# Supplementary material for: Psychometric Properties of the Chinese SUPPS-P Impulsive Behavior Scale: Factor Structure and Measurement Invariance Across Gender and Age
Source: Front Psychiatry. 2020 Nov 19;11:529949. doi: 10.3389/fpsyt.2020.529949 (PMC7710909; doi:10.3389/fpsyt.2020.529949)
Supplement: Supplementary file 3 [file Table_3.DOCX]

**Supplemental Table 3**

The original French version of the SUPPS-P

Vous trouverez ci-dessous un certain nombre d'énoncés décrivant des manières de se comporter ou de penser. Pour chaque affirmation, veuillez indiquer à quel degré vous êtes d'accord ou non avec l'énoncé. Si vous êtes Tout à fait d'accord avec l'affirmation encerclez le chiffre 1, si vous êtes Plutôt d'accord encerclez le chiffre 2, si vous êtes Plutôt en désaccord encerclez le chiffre 3 et si vous êtes Tout à fait en désaccord encerclez le chiffre 4. Assurezvous que vous avez indiqué votre accord ou désaccord pour chaque énoncé ci-dessous.

|  | Items  (French) | Tout à fait  d'accord | Plutôt  d'accord | Plutôt en  désaccord | Tout à fait  en  désaccord |
| --- | --- | --- | --- | --- | --- |
| 1 | D’habitude je réfléchis soigneusement avant de faire quoi que ce soit. | 1 | 2 | 3 | 4 |
| 2 | Quand je suis vraiment enthousiaste, j’ai tendance à ne pas penser aux conséquences de mes actions. | 1 | 2 | 3 | 4 |
| 3 | J’aime parfois faire des choses qui sont un petit peu effrayantes. | 1 | 2 | 3 | 4 |
| 4 | Quand je suis contrarié(e), j’agis souvent sans réfléchir. | 1 | 2 | 3 | 4 |
| 5 | Je préfère généralement mener les choses jusqu’au bout. | 1 | 2 | 3 | 4 |
| 6 | Ma manière de penser est d’habitude réfléchie et méticuleuse. | 1 | 2 | 3 | 4 |
| 7 | Quand la discussion s’échauffe, je dis souvent des choses que je regrette ensuite. | 1 | 2 | 3 | 4 |
| 8 | J’achève ce que je commence. | 1 | 2 | 3 | 4 |
| 9 | J’éprouve du plaisir à prendre des risques. | 1 | 2 | 3 | 4 |
| 10 | Quand je suis ravi(e), je ne peux pas m’empêcher de m’emballer. | 1 | 2 | 3 | 4 |
| 11 | Une fois que je commence un projet, je le termine presque toujours. | 1 | 2 | 3 | 4 |
| 12 | J’aggrave souvent les choses parce que j’agis sans réfléchir quand je suis contrarié(e). | 1 | 2 | 3 | 4 |
| 13 | D’habitude je me décide après un raisonnement bien mûri. | 1 | 2 | 3 | 4 |
| 14 | Je recherche généralement des expériences et sensations nouvelles et excitantes. | 1 | 2 | 3 | 4 |
| 15 | Quand je suis vraiment enthousiaste, j'agis souvent sans réfléchir. | 1 | 2 | 3 | 4 |
| 16 | Je suis une personne productive qui termine toujours son travail. | 1 | 2 | 3 | 4 |
| 17 | Quand je me sens rejeté(e), je dis souvent des choses que je regrette par la suite. | 1 | 2 | 3 | 4 |
| 18 | Je me réjouis des expériences et sensations nouvelles même si elles sont un peu effrayantes et non-conformistes. | 1 | 2 | 3 | 4 |
| 19 | Avant de me décider, je considère tous les avantages et inconvénients. | 1 | 2 | 3 | 4 |
| 20 | Quand je suis très heureux/heureuse, j’ai l’impression qu’il est normal de céder à ses envies ou de se laisser aller à des excès. | 1 | 2 | 3 | 4 |

**Supplemental Table 4**

The translated Chinese version of the SUPPS-P

下面是一些描述行为或思考方式的陈述。对于每一个陈述，请说明你在多大程度上同意或不同意。如果完全同意，请勾选数字1，如果你部分同意，请勾选数字2，如果你部分不同意，请勾选数字3，如果你完全不同意，请勾选数字4。请务必填写全部题目。

|  | Items  (Chinese) | 完全  同意 | 部分  同意 | 部分  不同意 | 完全  不同意 |
| --- | --- | --- | --- | --- | --- |
| 1 | 我通常在做任何事之前都会仔细思考。 | 1 | 2 | 3 | 4 |
| 2 | 当我处于很兴奋的状态时，我通常不会去思考自己行为的后果。 | 1 | 2 | 3 | 4 |
| 3 | 我有时喜欢做让人有点害怕的事儿。 | 1 | 2 | 3 | 4 |
| 4 | 当我心烦时，我经常不经思考就行动。 | 1 | 2 | 3 | 4 |
| 5 | 总的来说，我做事会有始有终。 | 1 | 2 | 3 | 4 |
| 6 | 我的想法通常比较谨慎，有一定的目的性。 | 1 | 2 | 3 | 4 |
| 7 | 在争论激烈的时候，我经常会说一些后来让自己后悔的话。 | 1 | 2 | 3 | 4 |
| 8 | 我做事会有始有终。 | 1 | 2 | 3 | 4 |
| 9 | 我非常喜欢有冒险性的活动。 | 1 | 2 | 3 | 4 |
| 10 | 当我感觉开心时，我常会感到自己的行为有些失控。 | 1 | 2 | 3 | 4 |
| 11 | 我一旦开始做一件事，就会持之以恒的做下去。 | 1 | 2 | 3 | 4 |
| 12 | 因为在心烦时会不加思考而行动，我经常会把事情变得更糟。 | 1 | 2 | 3 | 4 |
| 13 | 我通常通过仔细推理才做出决定。 | 1 | 2 | 3 | 4 |
| 14 | 我通常会寻求新鲜刺激的感觉和经历。 | 1 | 2 | 3 | 4 |
| 15 | 当我感到很兴奋时，我通常做事不假思索。 | 1 | 2 | 3 | 4 |
| 16 | 我是个工作很有成效的人，总是能把工作做完。 | 1 | 2 | 3 | 4 |
| 17 | 当我感到被否定或被排挤时，我经常会说些让自己将来后悔的话。 | 1 | 2 | 3 | 4 |
| 18 | 我欢迎新鲜刺激的感觉和经历，即使它们有点吓人或是不合传统。 | 1 | 2 | 3 | 4 |
| 19 | 下定决心之前，我会考虑所有的有利条件和不利条件。 | 1 | 2 | 3 | 4 |
| 20 | 当我特别开心的时候，我会觉得放纵下自己，随心所欲，做些出格的事儿也没什么。 | 1 | 2 | 3 | 4 |

Cotation: [R] = items à renverser 计分：[R] = 反向计分项目

Urgence: 4[R]; 7[R]; 12[R]; 17[R]; 急迫性：4[R]; 7[R]; 12[R]; 17[R];

Urgence positive: 2[R]; 10[R]; 15[R]; 20[R]; 正性急迫性：2[R]; 10[R]; 15[R]; 20[R];

Manque de Préméditation: 1; 6; 13; 19; 缺乏预见性：1; 6; 13; 19;

Manque de Persévérance: 5; 8; 11; 16; 缺乏持久性：5; 8; 11; 16;

Recherche de Sensation: 3[R]; 9[R]; 14[R]; 18[R]. 感觉寻求：3[R]; 9[R]; 14[R]; 18[R].
